# Cognitive deficits associated with optic aphasia: Neuropsychological contribution to a differential diagnosis

**DOI:** 10.1590/S1980-57642009DN20200013

**Published:** 2008

**Authors:** Melissa de Almeida Rodrigues, Carla Cristina Adda, Mara Cristina de Souza Lucia, Milberto Scaff, Eliane Correa Miotto

**Affiliations:** 1 Neuropsychologist of the Psychology Division, Hospital das Clinicas, University of Sao Paulo, Brazil.; 2 Neuropsychologist of the Psychology Division, Hospital das Clinicas, University of Sao Paulo, Brazil.; 3 Director of the Psychology Division, Hospital das Clinicas, University of Sao Paulo, Brazil.; 4 Professor of the Neurology Department, Hospital das Clinicas, University of Sao Paulo, Brazil.

**Keywords:** optic aphasia, neuropsychological assessment, differential diagnosis

## Abstract

Optic aphasia is characterized by a deficit in naming objects presented visually, as a result of left occipito-temporal lesion. It differs from other neuropsychological disorders due to the nature of the deficits and impairment of cognitive function. A 52 year-old patient, admitted after an episode of sub-acute infarction in the territory of the left posterior cerebral artery involving the temporo-occipital region, was submitted to neuropsychological evaluation as part of a diagnostic investigation and presented specific characteristics of this disorder, as well as impairment to episodic memory. The relevance of the present case is justified not only due to the rarity of the disorder, but also because it highlights the importance of differential diagnosis in the treatment of patients.

## Abstract (pt)

Afasia óptica consiste num déficit de nomear objetos apresentados visualmente como resultado de lesão occipito-temporal esquerda. Difere de outras desordens neuropsicológicas devido à natureza do déficit e comprometimento de funções cognitivas. Um paciente de 52 anos, internado após episódio de infarto subagudo em território de artéria cerebral posterior esquerda com acometimento da região têmporo-occipital, foi submetido à avaliação neuropsicológica como parte de investigação diagnóstica e apresentou características específicas desta desordem, bem como comprometimento de memória episódica. A relevância deste estudo é justificada não somente pela raridade com que a afasia óptica se apresenta, mas porque evidencia a importância do diagnóstico diferencial no tratamento dispensado aos pacientes.

Optical aphasia was first described in 1889 by Freud^1,2^ as a neuropsychological disorder that is chiefly characterized by a difficulty in naming objects presented visually. The dysfunction may be followed by right homonymous hemianopsia, alexia and disturbances in the visual identification of faces and colors.^3^ It is secondary to a lesion in the temporo-occipital territory and splenium of the callosal corpus, generated by infarct in the territory of the left posterior brain artery.^1,4,5^

In an attempt to describe this disorder, several theories have been put forward (Table 2). The superadditive impairments in vision and naming theory holds that, based on the fact that the left temporo-occipital region plays an important role in both visual and semantic processes,^2,5^ optic aphasia may be a result of lesions to the pathway that maps visual input to semantics, and the pathway that maps semantics to name responses, if the effects of theses lesions were cumulative.^2^ Thus, a task that requires both pathways (naming visually presented objects for instance) manifests a much higher error rate then the sum of errors in tasks involving one pathway or the other.

**Table 2:** Models of optic aphasia2.

Models	Theories	Limitations
Canonical Model	A visual processing system feeds its output into a semantic system which in turn feeds its output into a naming system. One cannot name a visually pre­sented object until one first knows what the object is.	One cannot place the lesion in vision, semantics, or the pathway connecting them, because patients can non-verbally demonstrate their recognition of visually presented objects. Neither can one place the lesion in naming or the pathway between seman­tics and naming, because patients are unimpaired in their ability to name objects presented in the tactile or auditory modalities.
Direct Visual Naming Pathway	There is a direct, uninterrupted pathway between vi­sion and naming. Optic aphasia results when the di­rect visual naming pathway becomes disconnected.	There are no documented cases of individuals who can name visual objects without any knowledge of what the objects are.
Modality-Specific Semantic Systems	Each modality has a corresponding semantic system. Optic aphasia arises when there is a disconnection between verbal semantics and visual semantics.	It does not explain the ability of optic aphasics. To sort visually dissimilar items into the same super­ordinate category.
Impaired Access to Semantics from Vision	There is an impairment in accessing a unified seman­tic system from vision. Whereas nonverbal responses may be initiated by activation of isolated semantic features from isolated visual features, naming re­quires access to a complete semantic representation.	Studies indicating poor performance on difficult nonverbal tasks may simply point to the fact that some patients indeed have a greater semantic deficit than others, apart from their inability to name visu­ally presented objects.
Hemisphere-Specific Semantic System	There is an independent semantic system for each hemisphere. Optic aphasia occurs when there is a disconnection between visual input and left hemi­sphere semantics	The major assumption behind this hypothesis - qualitatively distinct semantic for each hemisphere system - was questioned

Although optic aphasia is often associated with agnosia,^6,7^ it differs in the capacity to copy shapes – a compromised ability in aperceptive agnosia^3,8,9^ – and in recognition of the presented object through other sensorial means^10^ – also compromised in associative agnosia.^3,8,9,11^ Four important aspects must be considered to differentiate among these disorders:

1 – Patients with optical aphasia are able to demonstrate by gestures or mimes that, although they are unable to name a certain object, they recognize it.
2 – There are mistakes while naming the objects, however, these are semantically related to the presented object;
3 – There is insensitivity concerning the quality of visually presented stimulus such as drawings or tri-dimensional objects;
4 – There is no compromise in daily life activities.^2,6,12^

It is also important to discriminate optic aphasia from anomia, which presents as an incapacity to name objects based on their definitions, as well as objects that are presented through tactile and auditory means.^2^

Despite the differences described, the difficulty in identifying optical aphasia is common, not only because of its similarities to other pathologies, but also due to its rarity. The differential diagnosis is possible through a group of exams and evaluations performed by a multi-professional team which has the capacity to recognize the symptoms. Among these evaluations, the importance of the neuropsychological assessment is confirmed because this kind of evaluation attempts to determine which brain functions are particularly compromised, which are preserved and how the deficits influence behavior patterns displayed by the patient.^13^

## Case report

A.M.B., male, 52 years-old, truck driver and 11 years of education, was admitted in the emergency room after an episode of vision darkening and weakness in the inferior limbs that progressed to difficulty in naming people and objects after 12 hours. A CT scan revealed a hypodensity area in the left occipito-temporo region and MRI revealed subacute infarct in the left posterior cerebral artery territory. This had affected the temporo-occipital region, including the left hippocampus.

The preliminary neurological exam detected difficulty in identifying colors, reading words in spite of the preserved capacity of recognizing letters and writing, and right hemianopsia. Eye ground exams were normal, confirmed by ophthalmologic exam. According to the evaluation by a speech-language therapist, the patient displayed difficulty in naming objects; slow reading performance with presence of repetitions and prolongation; preserved writing and naming of pictures of action. The patient was subsequently submitted to neuropsychological evaluation comprising quantitative and qualitative tests.

During the neuropsychological evaluation, difficulties in naming visually presented objects, reading and episodic memory were observed (Table 1).

**Table 1:** Neuropsychological tests and results.

Models	Test	Results	Classification
**Object naming**			
Visual input	Boston Naming Test^16^	25/60	Severe impairment
Tactile input		15/15	Preserved
**Visual perception**			
Spatial perception and orientation	Judgment of Line Orientation^17^	22/30	Mild impairment
Visual form discrimination	Visual Form Discrimination^17^	29/32	Preserved
Visual-spatial and constructional	Clock Drawing Test^18^	15/15	Preserved
	Picture Copy		Preserved
Visual object and spatial perception	Visual Object and Space		
	Perception Batttery^19^		
	Incomplete Letters	19/20	Preserved
	Position Discrimination	20/20	Preserved
Visual-spatial ability	Hooper Visual Organization Test^20^		Severe impairment
Unfamiliar face recognition	Facial Recognition^17^	42/54	Preserved
**Intellectual abilities**	Wais III^21,22^	99	Preserved
**Memory**			
Short term	Digits (Wais III)^21^	14/30	Preserved
Long term	Hopkins Verbal Learning Test^23^		
	Total recall	17/36	Severe Impairment
	Delayed recall	0/12	Severe Impairment
	Recognition	0/12	Severe Impairment
	Brief Visualspatial Memory		
	Test^24^		
	Total recall	15/36	Moderate Impaiment
	Delayed recall	8/dez	Severe Impairment
	Recognition	4/6	Moderate Impaiment
	Face Recognition^25^	23/25	Preserved
**Abstract concepts**	Modified Card Sorting Test^26^	6/6	Preserved
**Language**			
Scene description	Thematic Picture^27^		Preserved
Reading skills			
Words		0/0	Reads with difficulties
Text			Guesses the end of the words
Spontaneous speech	Thematic Picture^27^		Preserved
Comprehension	Mini-Mental State Examination^28^	29/30	Preserved
Repetition	Mini-Mental State Examination^28^	29/30	Preserved

## Discussion

The location of the lesion and neuropsychological findings of this evaluation confirm the clinical suspicion that, concerning language skills, the patient showed specific difficulty in naming objects through visual presentation of stimulus. This confirmed suspected optic aphasia. However, this condition is similar to other pathologies suspected by the team during the period of diagnostic investigation. The first hypothesis, anomia, was refuted because the subject was able to name objects presented by other sensorial means.^9^

The difficulty in naming visually presented objects may be a consequence of perceptual disorder, observed in visual agnosia,^4,13^ however, because of the normal performance in visual-perceptive and visual-spatial tests, and semantic knowledge about the objects that he was unable to name, the hypothesis of aperceptive or associative agnosia was ruled out. The capacity for identifying shapes and faces through different points of view (object constancy) suggests that the difficulty in naming objects occurs even if the structural representations are preserved.^5^

The naming deficit could be attributed to some kind of aphasia, however, in the case of optic aphasia, the representations and processes that constitute language are intact as well as some aspects of language such as spontaneous speech, repetition and comprehension. The impairment is found in access to it,^5^ which also occurs in pure alexia, reported by various authors as a comorbidity to optic aphasia.^15^ In both cases the difficulty in reading words and texts is associated to a disconnection between occipital areas and language areas within the left hemisphere. The fact that the majority of optic aphasia cases are associated to a deficit in the right visual field^9^ explains the difficulty described by the patient in seeing the second half of the words. This difficulty was not specific to reading (the patient found watching TV difficult for the same reason), but it did not compromise his performance in copying pictures as well as in perception tests.

In relation to the affected area in the brain, although the patient did not show compromise in the splenium of the callosal corpus on imaging exams, we found reports in the literature with the same characteristics,^4^ which taken together with the clinical evidence and results of neuropsychological assessment, constitute a fundamental triad for the diagnosis of neuropsychological impairment.

Although it would be valuable to analyze a larger amount of data collected from other patients with this same disorder in order to obtain consistent results, including a more specific language evaluation, the relevance of this present case study is justified not only due to its rarity but also because it highlights the importance of differential diagnosis concerning patient treatment.
